# Defect in Sensing Human Thrombin by Porcine Endothelial Protease‐Activated Receptor‐1: Molecular Incompatibility Between Porcine PAR‐1 and Human Thrombin

**DOI:** 10.1111/xen.70041

**Published:** 2025-04-17

**Authors:** Thi Xoan Hoang, Ju‐Young Bang, Vinh Phuoc Nguyen, Phu Chi Vu, Ik Jin Yun, Hee Jung Kang, Jae Young Kim

**Affiliations:** ^1^ Department of Life Science Gachon University Seongnam Kyeonggi South Korea; ^2^ Department of Surgery Konkuk University School of Medicine Seoul South Korea; ^3^ Department of Laboratory Medicine Hallym University College of Medicine Anyang South Korea

**Keywords:** coagulation, inflammation, protease‐activated receptor‐1, thrombin, xenotransplantation

## Abstract

Xenotransplantation, the transplantation of organs from pigs to humans, presents significant challenges due to immune rejection, which is driven by molecular incompatibilities between species. This study investigates the compatibility between human thrombin and porcine protease‐activated receptor‐1 (PAR‐1), a key regulator of both coagulation and inflammatory responses. Human thrombin activates PAR‐1 in human vascular endothelial cells, but our results demonstrate that human thrombin does not effectively activate PAR‐1 in porcine vascular endothelial cells due to differences in amino acid sequences, particularly at the thrombin cleavage site and the Hir domain. Protein‐protein docking analysis further reveals that porcine PAR‐1 forms less stable interactions with human thrombin compared to human PAR‐1, resulting in reduced activation. This molecular incompatibility likely contributes to impaired nitric oxide (NO) production, endothelial dysfunction, and increased inflammation, which are critical for the survival of transplanted organs. Additionally, experiments using the PAR‐1 inhibitor vorapaxar (Vor) show that inhibiting PAR‐1 signaling can suppress inflammatory cytokine and chemokine expression in co‐cultures of human macrophages and porcine endothelial cells. These findings suggest that selective PAR‐1 inhibitors or targeted therapies regulating thrombin‐PAR‐1 signaling may improve the success rate of xenotransplantation. However, further in vivo studies are needed to validate these findings and explore therapeutic interventions targeting thrombin‐PAR‐1 interactions to enhance xenograft survival.

## 1 Introduction

Due to the shortage of organs needed for human transplantation, research on xenotransplantation—transplanting organs from pigs into humans—is increasing. However, strong immune rejection occurs due to the high immune incompatibility between pigs and humans [1]. Coagulation and inflammatory responses are major obstacles to successful xenotransplantation. Therefore, the compatibility between donor and recipient molecules, which play critical roles in blood coagulation and inflammatory responses, is essential for successful xenotransplantation.

Thrombin is a serine protease that cleaves specific N‐terminal amino acid sequences of G protein‐coupled receptors, playing a key role in the blood coagulation mechanism [2]. Its natural ligands include fibrinogen, PARs (protease‐activated receptors), thrombomodulin (TBM), platelet glycoprotein Ib, and heparin cofactor II. Among these, fibrinogen and heparin cofactor II are produced in the liver, so compatibility issues with recipient‐derived molecules, such as thrombin, do not arise during organ transplantation, except in the case of liver transplantation. Additionally, platelet glycoprotein Ib presents no compatibility issues since platelets are derived from the recipient. In contrast, TBM and PARs, which are expressed in vascular endothelial cells, originate from the organ donor, necessitating consideration of their molecular compatibility with thrombin.

TBM is expressed on the surface of vascular endothelial cells, where it binds to thrombin and alters its substrate specificity to facilitate the activation of protein C, thereby playing a role in anticoagulation. Previous studies have shown that porcine TBM binds to human thrombin but is ineffective in activating protein C, due to structural differences between porcine TBM and human thrombin. This incompatibility can result in thrombus formation and tissue damage within the transplanted organ [3]. However, the molecular compatibility between porcine vascular endothelial cell PARs and human thrombin has not been studied.

Thrombin activates three types of PARs (PAR‐1, PAR‐3, and PAR‐4), while PAR‐2 is activated by trypsin [4]. PARs play a significant role in hemostasis. PAR‐1 activates coagulation factors IXa, Xa, and the VIIa/TF complex, promoting various stages of the coagulation process [5, 6], especially in the early stages of platelet aggregation during hemostasis [4, 7]. PAR‐3 primarily acts as a cofactor for PAR‐4, enhancing platelet aggregation [8, 9]. PAR‐4 is activated by thrombin in humans and contributes to the hemostasis process by promoting platelet activation [7, 9].

In addition to their roles in coagulation, PAR receptors also mediate inflammatory responses, revealing a close connection between coagulation and inflammation [10, 11]. PAR‐1 contains a hirudin‐like domain with a high‐affinity thrombin binding site, allowing thrombin to efficiently activate PAR‐1 [12]. Thrombin‐mediated activation of PAR‐1 leads to a cascade of intracellular events, including calcium influx and the activation of protein kinase C, which subsequently triggers the release of calcium from the sarcoplasmic reticulum into the cytoplasm [13, 14]. PAR‐1 modulates vascular tone by mediating thrombin‐induced biphasic phosphorylation of endothelial nitric oxide synthase (eNOS), which controls vascular tone through nitric oxide (NO) production [15]. Activation of PAR‐1 also leads to the activation of MAPKs (mitogen‐activated protein kinases), increasing endothelial barrier permeability [16]. Furthermore, PAR‐1 activation promotes the expression of inflammatory cytokines in vascular endothelial cells and leukocytes [11, 17], as well as chemotactic proteins like MCP‐1 and RANTES [18, 19] and cell adhesion molecules like selectins, VCAM‐1, and ICAM‐1, which enhance immune cell migration to the inflammation site [17, 20]. This also increases the permeability of vascular endothelial cells, allowing plasma components to infiltrate the inflammation site [13, 14]. These findings highlight the crucial regulatory roles that PAR‐1 plays in hemostasis and inflammatory responses.

In this study, to investigate the functional compatibility of human thrombin with porcine PAR‐1, we treated porcine arterial vascular endothelial cells with human thrombin in the presence or absence of vorapaxar (Vor), a PAR‐1‐specific inhibitor. We assessed intracellular calcium levels (Ca^2+^), NO levels, inflammatory cytokine/chemokine gene expression, and endothelial permeability. Additionally, we compared the amino acid sequences of human and porcine PAR‐1 to confirm their functional compatibility.

## 2 Materials and Methods

### 2.1 Reagents and Antibodies

Thrombin from human plasma was purchased from Chrono‐Log (Cat#: 386), PAR‐1 antagonist Vor from SPL Life Science, South Korea (Cat#: 618385‐01‐6). Anti‐p65 NF‐kB antibody (PA5‐16545) and anti‐p‐PAR‐1 antibody (PA5‐105095) were obtained from Thermo Fisher Scientific. Anti‐PAR‐1 antibody (C11, sc‐271100), anti‐β‐actin antibody (sc‐47778), horseradish peroxidase‐conjugated anti‐mouse IgG antibody (sc‐2055), or anti‐goat IgG antibody (sc‐2020) were from Santa Cruz. Brilliant violet 480‐conjugated anti‐CD141 (TBM) antibody was purchased from DB Bioscience San Jose, CA, USA (Clone1A4, Cat#: 746604).

### 2.2 Cell Culture

The human umbilical vein endothelial cell line (HUVEC) and porcine aortic endothelial cell line (PAOEC) were purchased from Lonza bioscience, Basel, Switzerland. HUVECs were cultured in endothelial growth medium EGM‐2 (Lonza) supplemented with 1% antibiotic‐antimycotic (Invitrogen Corporation), PAOECs were cultured in Dulbecco's Modified Eagle's Medium (DMEM) added with 10% heat‐inactivated fetal bovine serum (FBS) and 1% antibiotic‐antimycotic (Invitrogen Corporation).

Human monocyte derived from patient with acute monocytic leukemia (THP‐1) (Korean cell line bank, Seoul, South Korea) was cultured in RPMI‐1640 media (Welgene Inc., Daegu, South Korea) added with 10 mM HEPES buffer (Invitrogen Corp., Gibco BRL, MD, USA), β‐mercaptoethanol (Invitrogen Corp.) supplemented with 10% heat‐inactivated FBS and 1% antibiotic‐antimycotic. Cells were maintained in a humidified incubator containing 5% CO_2_ at 37°C.

### 2.3 RNA Preparation and Real‐Time Quantitative PCR

After treatment with chemicals in different experiments, total RNA from culture plates was isolated by easy‐BLUE^TM total RNA Extraction Kit (iNtRON Biotechnology, Inc.) following to manufacturer's instructions. RNA concentration was quantified by MaestroNano MicroVolume Spectrophotometer (Maestrogen, Las Vegas, NV, USA). cDNA was synthesized from 2 µg of total RNA using Hyperscritp 2 X RT Master Mix (Geneall Biotechnology). Real‐time PCR of targeted genes was performed by using QuantiSpeed SYBR NO‐ROX kit (PhileKorea) in a Rotor‐gene system (Quiagen). PCR amplification was performed using the following primer sets: TNF‐α 5′‐TGAGCACTGAAAGCATGATCC‐3′, 5′‐ GGAGAAGAGGCTGAG GAACA‐3′, IL‐1β 5′‐GGGATAACGAGGCTTATGTGC‐3′, 5′‐AGGTGGAGAGCTTTCAGTTCA‐3′, IL‐6 5′‐GACCCAACCACAAATGCCAG‐3′, 5′‐GAGTTGTCATGTCCTGCAGC‐3′, hPAR‐1 5′‐CATCTGTGTACACCGG‐3′, 5′‐TGCCAATCACTGCC‐3′, hPAR‐2 5′‐GGTCCTCAGATGGGAATTGC‐3′, 5′‐GGAGCTCCTAGCAATCCTG‐3′, hMCP‐1 5′‐CCCAAGAATCTGCAGCTAAC‐3′, 5′‐GGTAGAACTGTGGTTCAAGAGG ‐3′, hRANTES 5′‐CCTCATTGCTACTGCC‐3′, 5′‐GCAAGCAGAAACAGGC‐3′, hMIP‐1 5′‐GTGTGACCTCCACAGCTAC‐3′, 5′‐CTGCCTACACAGGCTGATGA‐3′, hTF 5′‐GGGCTGACTTCAATCCATGT‐3′, 5′‐GAAGGTGCCCAGAATACCAA‐3′, pPAR‐1 5′‐CATTTCCGGACGTGGAT‐3′, 5′‐TAGAGGAAGGCTTACGAG‐3′, pPAR‐2 5′‐GAGGTTATGCACGAGG‐3′, 5′‐GACACTTCCAGCAAGCC‐3′, pMCP‐1 5′‐CCGAGAATCTGAAGAC‐3′, 5′‐GAGGACTGAGATTCAC‐3′, pRANTES 5′‐GCTCCATGGCAGCAG‐3′, 5′‐GCAAGAAGCAGTAGG‐3′, pMIP‐1 5′‐GTCTTCTCTGCACCACTTG‐3′, 5′‐GGTCAGAGATGTATTCCTGG‐3′, pTF 5′‐TGGGAGTTGGTGGACTTTTC‐3′, 5′‐CATCAACAGCCTTCAGGTCA‐3′ and GAPDH 5′‐ACAGCCTCAAGATCATCAGCAAT‐3′, 5′‐AGGAAATGAGCTTGACAAAGTGG‐3′. Sample normalization was performed using the human β‐actin gene as an endogenous control. For each sample, the relative abundance of target mRNA was calculated from the C△t values of the target and endogenous β‐actin reference genes using the 2−△△cycle threshold (Ct) method.

### 2.4 Measurement of Intracellular Ca^2+^

To determine the Ca^2+^, 1×10^5^ cells/well were cultured in six‐well culture plates. After incubation, the cells were collected and washed with calcium‐free DPBS (Sigma‐Aldrich). Then, the cells were cultured with 50 µL Flou‐4‐NW‐dye for 30 min at 37°C in a state of blocking the light, followed by incubation at 25°C for 30 min. The fluorescence was measured by fluorescence microscopy (Olympus, CKX53, Tokyo, Japan) or by Cytomics FC500 MLP.

### 2.5 Immunofluorescence

After treatments, differentiated cells were fixed with 4% formaldehyde in PBS. Subsequently, the cells were blocked with 2% BSA in PBS. The cells were then incubated with the BV480‐conjugated anti‐TBM antibody for 45 min. The cell nucleus was stained with 10 µM Hoechst33342 for 10 min. The images were obtained by fluorescent microscope (Olympus, CKX53, Tokyo, Japan) and analyzed by Image J software.

### 2.6 Measurement of No Production

HUVECs or PAOECs were seeded at a density of 1×10^5^ cells per well in 24‐well plates. Following treatment, the media were collected from each well and centrifuged at 10 000 × *g* for 1 min. The supernatant was harvested, and NO levels were measured using a total NO assay kit (BM‐NIT‐200 BIMAX Inc, Seoul, South Korea) according to the manufacturer's instructions. Absorbance was measured at 450 nm.

### 2.7 Western Blot

Treated cells were lysed with the Triton X‐100 lysis buffer and incubated for 15 min at 4°C before centrifugated at 14 000 RPM for 15 min at 4°C. Denatured proteins were run on SDS‐PAGE and blotted onto a polyvinylidene difluoride membrane. The membranes were blocked with 5% BSA, followed by probing with the following primary antibodies against p65 NF‐kB, PAR‐1; p‐PAR‐, and β‐actin. The membranes were then incubated with horseradish peroxidase‐conjugated anti‐goat IgG antibody (sc‐2020), or anti‐mouse IgG antibody (sc‐2055), and visualized with the ECL solution using the ChemiDoc MP system (Bio‐Rad) according to the manufacturer's protocol. β‐actin was used as a positive control for sample normalization.

### 2.8 Endothelial Cell Permeability Assay

To measure the permeability of endothelial cell monolayer, gelatin‐coated transwell with 0.4 µm pore was used in 24 well plates (Corning. Costar, The Netherlands). Briefly, HUVECs or PAOEC were plated at a density of 1 × 10^5^ cells per insert and were cultured for 48 h until the formation of a tight monolayer. The cells were washed, and 2 U/mL of thrombin were treated for 3 h. Then the cells were washed twice with PBS, followed by the addition of Horseradish Peroxidase substrate (HRP) (Thermo Fisher Scientific) into the top of the chamber where the cells were cultured. The supernatant in the lower chamber was collected at different time points (15, 30, 45, and 60 min), and the endothelial permeability was measured by the leakage of HRP using an ELISA reader at 450 nm.

### 2.9 Retrieval and Processing of Protein Structures

The 3D protein structures of human and porcine PAR‐1 were obtained from the AlphaFold Protein Structure Database (https://alphafold.ebi.ac.uk/) with the ID codes AF‐P25116‐F1 and AF‐A0A286ZU96‐F1, respectively. The 3D protein structure of human thrombin was retrieved from the Protein Data Bank (https://www.rcsb.org/) with the ID: 4az2. After downloading in PDB format, these three protein structures were processed by removing water molecules and heteroatoms (hetatm) using BIOVIA Discovery Studio Visualizer version 2021.

### 2.10 Protein‐Protein Docking Prediction

Protein‐protein docking for human thrombin with both human PAR‐1 and porcine PAR‐1 was performed using the HDOCK webserver. The docking parameters were kept at default settings, except for the selection of residue number 41 in the “Receptor binding site residue(s)” within the “Advanced Options” section. Residue 41, an Arginine amino acid in both human and porcine sequences, is the cleavage site of thrombin on PAR‐1. By specifying this residue, the docking process was directed to focus on this site and its neighboring regions, including the tethered ligand region and the hirudin‐like region (Hir). The HDOCK webserver is a highly integrated tool that combines homology search, template‐based modeling, structure prediction, macromolecular docking, biological information incorporation, and job management for robust and efficient protein‐protein docking. It employs a hybrid algorithm that integrates both template‐based and template‐free docking methods. Additionally, the HDOCK webserver offers a competitive advantage in terms of faster result generation compared to other protein docking web servers [21]. Among the top 10 binding models generated by the HDOCK webserver, “Model 1” achieved the highest Docking Score and Confidence Score, making it the best model. This structure was selected for further interaction analysis using the PDBsum webserver.

### 2.11 Statistical Analysis

The experiments were repeated at least three times and performed independently as mean ± SD. To compare the differences between various groups, one‐way analysis of variance (ANOVA) followed by post hoc test using SPSS 12.0 for Windows was applied. The significant differences were performed under *p* value < 0.05.

## 3 Results

### 3.1 PAR‐1 Inhibition Reduces the Levels of Inflammatory Cytokine and Chemokine Gene Expression Increased by the Co‐Culture of Human Macrophages and Pig Vascular Endothelial Cells

**FIGURE 1:**
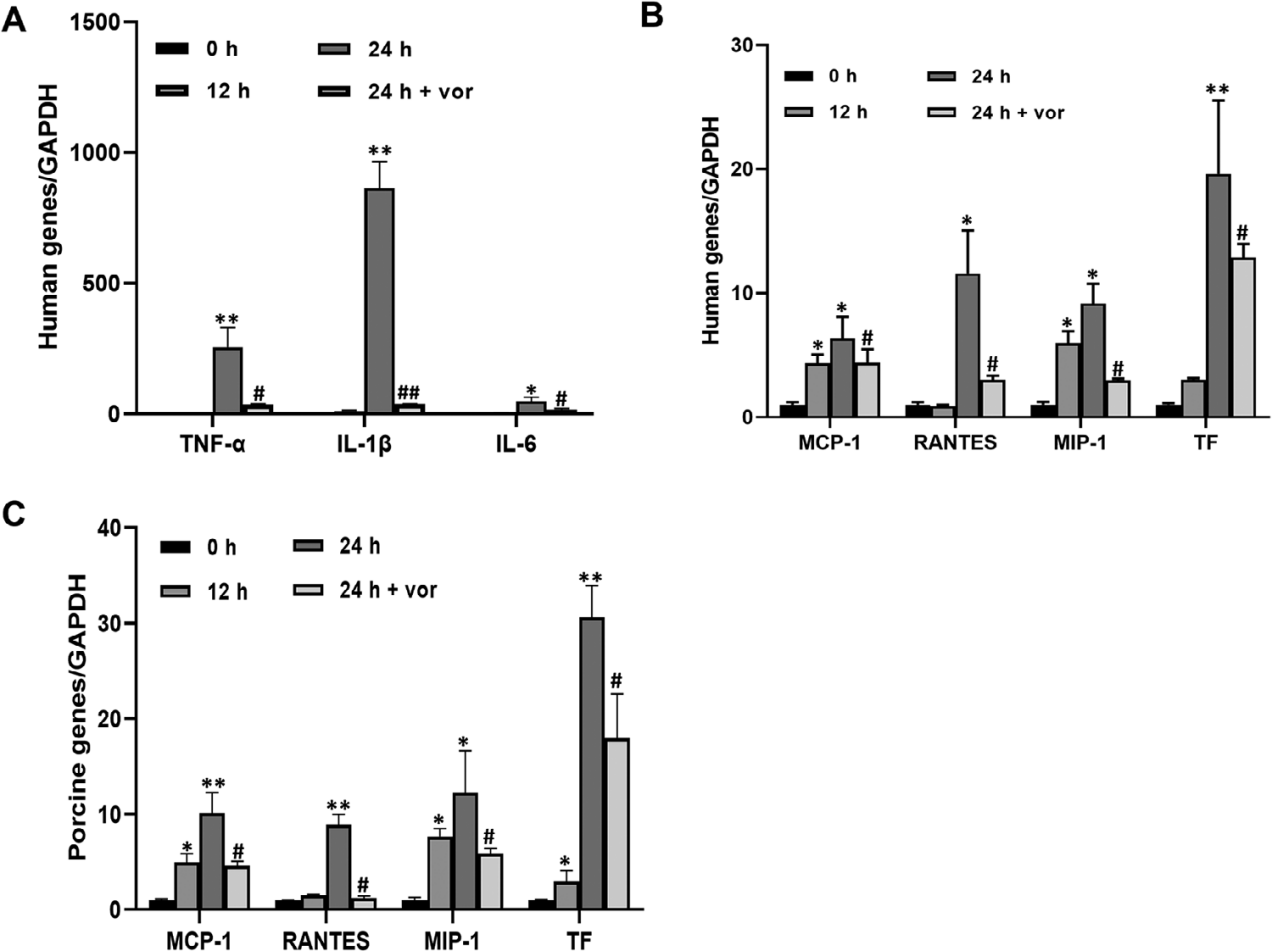
PAR‐1 antagonist suppresses the enhanced mRNA expression of pro‐inflammatory cytokines, chemokines, and tissue factors in human monocyte and porcine endothelial cell co‐culture. Porcine aortic endothelial cells (PAOEC) and human monocyte THP‐1 cells were co‐cultured for 12 or 24 h in the presence or absence of 100 nM PAR‐1 antagonist vorapaxar (Vor), which was added at the initial time point of co‐culture. mRNA levels of human pro‐inflammatory cytokines (A), chemokines and TF (B), and porcine chemokines and tissue factor (C) were examined using qRT‐PCR. *p < 0.05 versus 0 h group; **p < 0.01 versus 0 h group; #*p* < 0.05 versus 24 h group.

Vascular endothelial cells and macrophages are the major cells expressing PAR‐1 [22], and there is a possible cross‐talk between macrophages and vascular endothelial cells in inflammatory responses in pig‐to‐primate organ transplantation [23]. Therefore, to determine whether inhibiting PAR‐1 in these cells affects the expression of inflammatory mediators, we co‐cultured THP‐1 and PAOECs with or without the PAR‐1 inhibitor Vor. We then examined the mRNA expression levels of inflammatory cytokines TNF‐α, IL‐1β, IL‐6, chemokines MCP‐1, RANTES, MIP‐1, and tissue factor (TF) using human and pig gene‐specific primers, respectively. After 24 h of co‐culture, the mRNA expression levels of inflammatory cytokines in humans (Figure 1A) and chemokines and TF (Figure 1B) dramatically increased. The increased mRNA expression of inflammatory molecules was significantly suppressed by 100 nM Vor (Figure 1A,B). Similarly, the mRNA expression levels of porcine TF and inflammatory chemokines and TF increased by co‐culture were significantly suppressed by Vor treatment (Figure 1C).

### 3.2 Human Thrombin Increases Ca^2+^ and NO Levels in HUVECs but Not in PAOECs, and These Increases Are Mediated by PAR‐1

**FIGURE 2:**
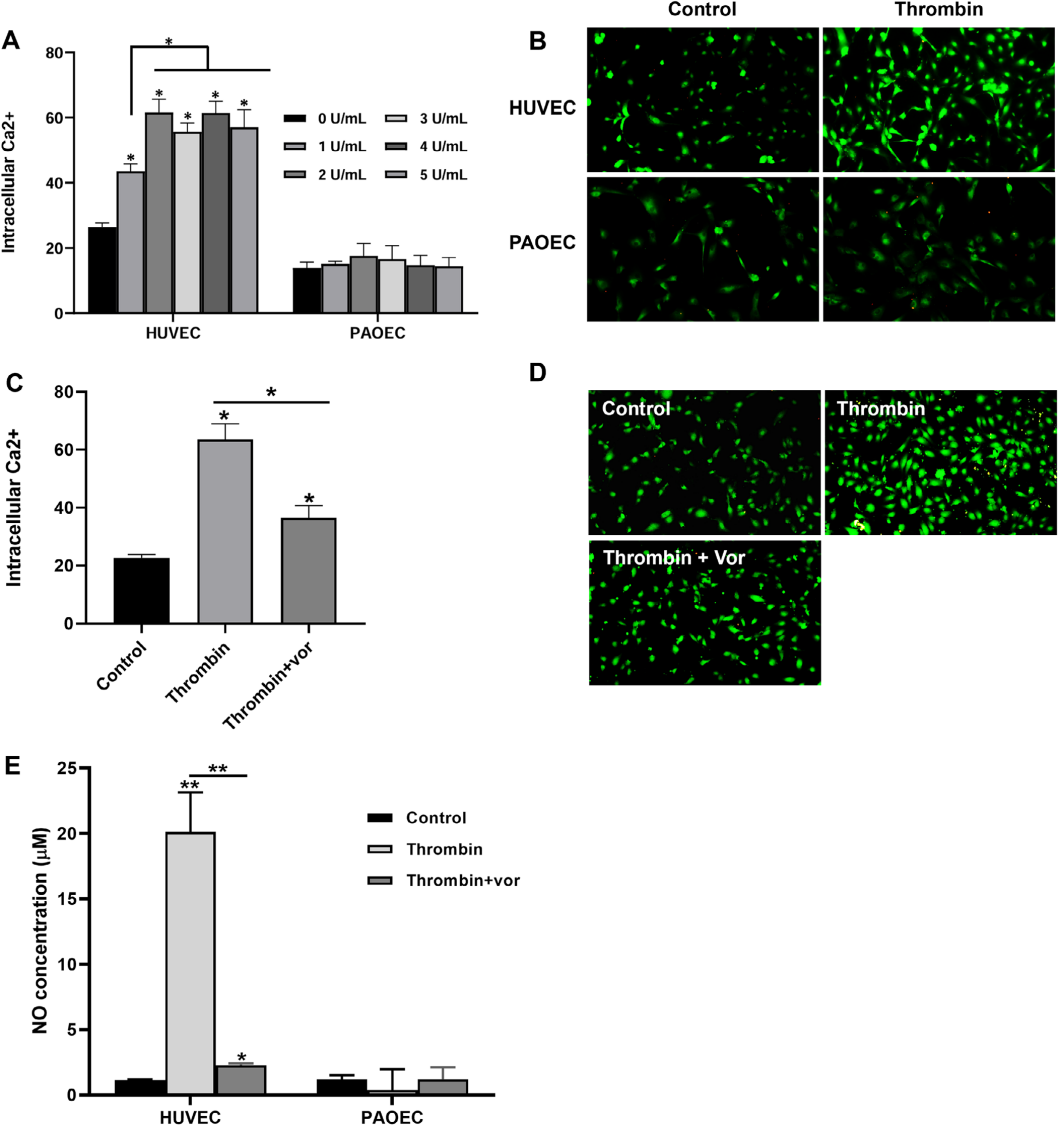
PAR‐1 antagonist suppresses human thrombin‐enhanced intracellular Ca^2+^ and nitric oxide levels in HUVECs but not PAOECs. HUVECs or PAOECs were treated with different concentrations of human thrombin for 2 h and intracellular levels of Ca^2+^ were measured by flow cytometry (A). Cells were treated with 2 U/mL human thrombin and Ca^2+^ levels were determined using a fluorescence microscope at 200 × magnification (B). HUVECs were treated with 2 U/mL of human thrombin and 100 nM vorapaxar (Vor) for 2 h (C, D) and Ca^2+^ levels were measured by flow cytometry (C) and fluorescence microscope at 200 × magnification (D). HUVECs or PAOCEs were treated with 2 U/mL of human thrombin for 2 h in the presence or absence of 100 nM PAR‐1 antagonist Vor. The supernatant was collected and nitric oxide level was measured (E). *p < 0.05, **p < 0.01 versus control group.

Calcium ions in vascular endothelial cells play an essential role in the production of NO, which promotes vasodilation, anti‐inflammatory, and antithrombotic effects, thereby maintaining vascular homeostasis. Additionally, since thrombin promotes NO production via PAR‐1 [15], we investigated intracellular calcium and NO production in response to human thrombin through PAR‐1 in HUVECs and PAOECs in the presence or absence of Vor. Figure 2 shows the typical changes in Ca^2+^ when HUVECs and PAOECs were exposed to various concentrations of thrombin. Dose‐response diagrams showed that thrombin‐induced maximum Ca^2+^ increase in HUVECs at 2 U/mL, whereas no significant changes in Ca^2+^ levels were observed in PAOECs with any amount of thrombin input (Figure 2A). Similarly, immunofluorescence staining of Ca^2+^ (green) in HUVECs and PAOECs also showed changes in intracellular Ca^2+^ levels. Microscopic images showed that thrombin‐induced intracellular Ca^2+^ increased in HUVECs, whereas no change was detected in PAOECs (Figure 2B).

In addition to the increase in intracellular Ca^2+^, thrombin also induced a significant elevation in NO levels in HUVECs. Thrombin caused a dose‐dependent increase in Ca^2+^ and NO levels, which were both attributed to PAR‐1 activation [15, 24]. To confirm that the increase in intracellular Ca^2+^ and NO levels in HUVECs was mediated by PAR‐1, we tested the effect of the PAR‐1 inhibitor Vor. Treatment of HUVECs with 2 U/mL of human thrombin increased both intracellular Ca^2+^ and NO levels, but these increases were significantly suppressed by treatment with 100 nM Vor (Figure 2C,E). In contrast, thrombin treatment did not cause any significant changes in Ca^2+^ and NO levels in PAPEC (Figure 2A,E). Similarly, the increased immunofluorescent signal of Ca^2+^ (green) in HUVECs induced by human thrombin was significantly reduced by treatment with 100 nM Vor (Figure 2D). This suggests that the increase in intracellular Ca^2+^ and NO levels induced by human thrombin is mediated via PAR‐1 activation by human thrombin.

### 3.3 Human Thrombin Increases the Expression Levels of Inflammatory Chemokine Genes in HUVECs but Not in PAOECs, and This Is Mediated by PAR‐1

**FIGURE 3:**
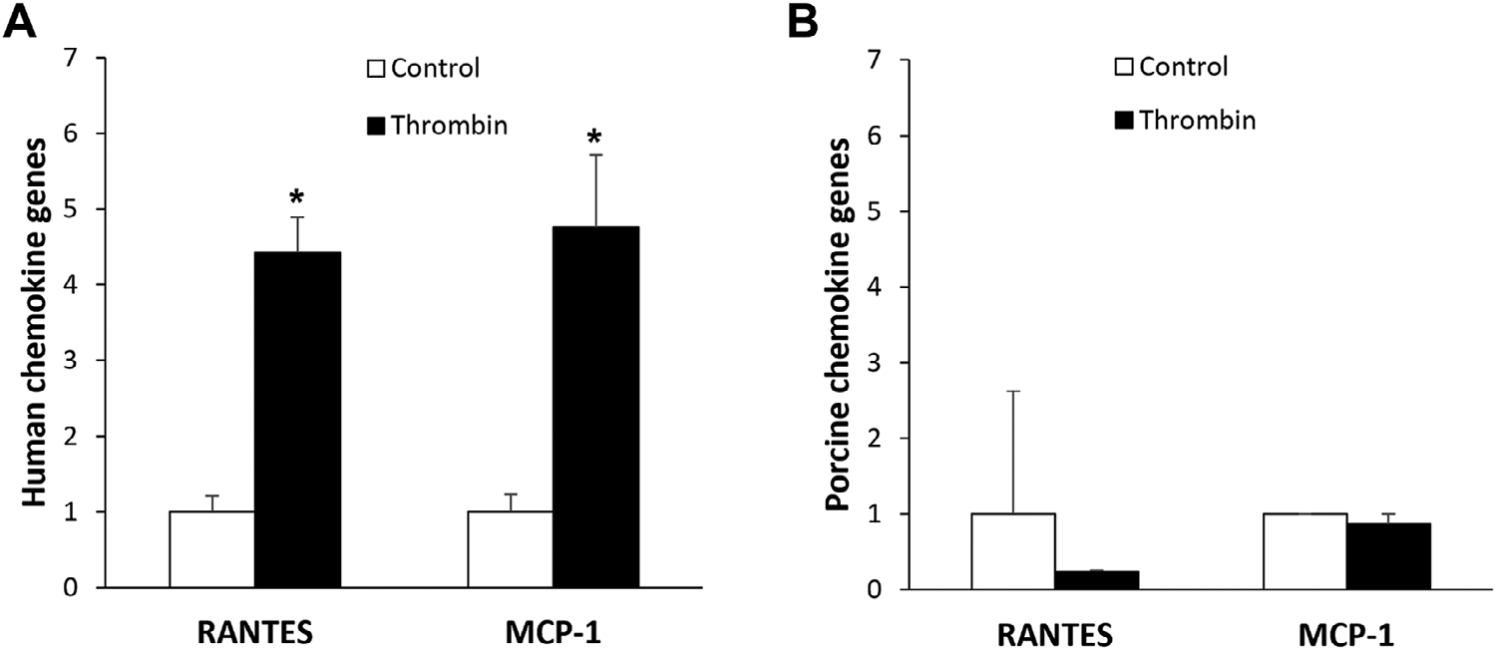
Differential chemokine expression between HUVEC and PAOEC in response to human thrombin. HUVECs or PAOECs were treated with 2 U/mL of human thrombin for 2 h. After cell harvesting levels of human chemokine (hRANTES, hMCP‐1) or porcine chemokine (pRANTES, pMCP‐1) mRNA expression were determined using qRT‐PCR. *p < 0.05 versus control group.

**FIGURE 4:**
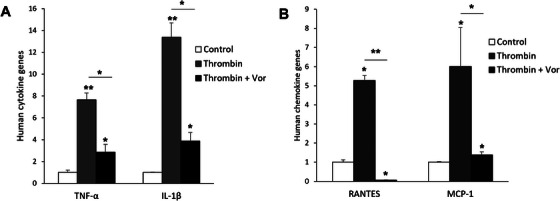
PAR‐1 antagonist suppresses human thrombin‐enhanced cytokine expression in HUVEC. HUVECs were treated with 2 U/mL of human thrombin and 100 nM vorapaxar (Vor) for 2 h and mRNA expression levels of human pro‐inflammatory cytokines (A) and chemokines (B) were measured by qRT‐PCR. *p < 0.05, **p < 0.005 versus control group.

Thrombin induces biological actions by activating PARs and the PAR‐1 activation is associated with pro‐inflammatory effects in different types of cells [11]. Therefore, human thrombin was applied to HUVECs and PAOECs, and the mRNA levels of the inflammatory chemokines MCP‐1 and RANTES were examined using quantitative real‐time PCR. In HUVECs, the expression of both RANTES and MCP‐1 mRNA was significantly increased by 2 U/mL of human thrombin, reaching approximately four times the level of the untreated control (Figure 3A). In contrast, in PAOECs, the expression levels of RANTES and MCP‐1 mRNA remained low regardless of human thrombin treatment (Figure 3B). The study investigated whether the expression of inflammatory cytokines and chemokines in HUVECs is increased by human thrombin, and if so, whether this increased expression of inflammatory molecules is reversed by a PAR‐1 specific inhibitor. When HUVECs were treated with 2 U/mL of human thrombin, the mRNA expression levels of the inflammatory cytokines TNF‐α and IL‐1β (Figure 4A) and the chemokines RANTES and MCP‐1 (Figure 4B) were significantly increased. However, when treated with 100 nM Vor, the elevated mRNA expression of cytokines and chemokines was markedly suppressed (Figure 4). This indicates that the increased expression of inflammatory cytokines and chemokines induced by human thrombin is related to the activation of PAR‐1 by human thrombin.

### 3.4 Human Thrombin Increases Endothelial Permeability in HUVECs but Not in PAOECs, and This Is Mediated by PAR‐1

**FIGURE 5:**
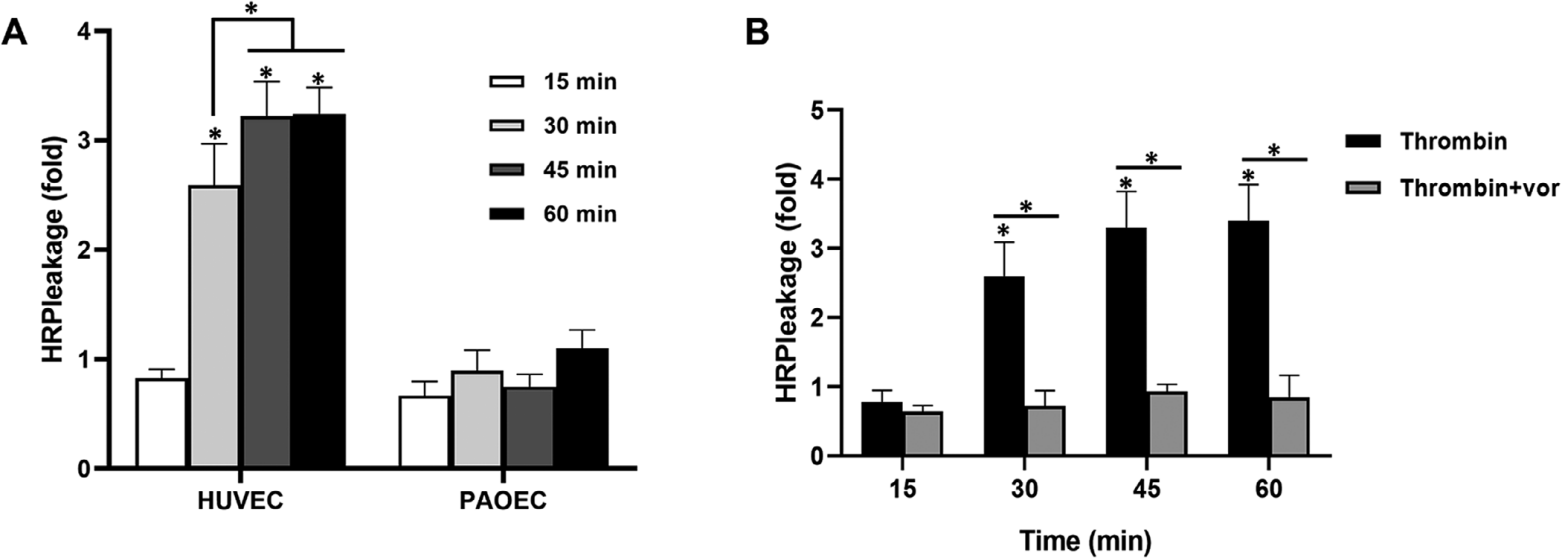
Differential endothelial permeability between HUVEC and PAOEC in response to human thrombin. HUVECs or PAOECs were seeded on 24‐well inserts. HRP was added to the upper chamber of the culture and the endothelial leakage was determined by measuring HRP levels in the lower chamber at different time points using an ELISA. Cells were treated with 2 U/mL of human thrombin for 3 h (A). Cells were treated with 2 U/mL of human thrombin for 3 h in the presence or absence of 100 nM PAR‐1 antagonist vorapaxar (Vor) (B). *p < 0.05 versus control group.

Thrombin activates the PAR‐1 receptor, disrupts adherent junctions, changes the shape of endothelial cells, and increases endothelial cell permeability [25, 26]. To confirm thrombin‐induced endothelial cell permeability, HUVECs and PAOECs grown on 0.4 µm pore filters were treated with human thrombin. The movement of HRP across the filters was then measured at various times. As shown in Figure 4A, the addition of human thrombin induced the movement of HRP across the endothelial monolayer in HUVECs by about 3.5 times. In contrast, there was no significant effect on PAOECs except at the 1‐h mark (Figure 5A). To confirm that the increased permeability level caused by human thrombin treatment in HUVECs was due to PAR‐1, we tested the effect of the Vor. Treatment of HUVECs with 2 U/mL of human thrombin increased permeability in a time‐dependent manner, reaching a level about 3–4 times higher, and this increased permeability was significantly suppressed to basal levels by treatment with 100 nM Vor (Figure 5B).

### 3.5 Thrombin Promotes PAR‐1 Phosphorylation, Which Is Inhibited by the PAR‐1 Antagonist

**FIGURE 6:**
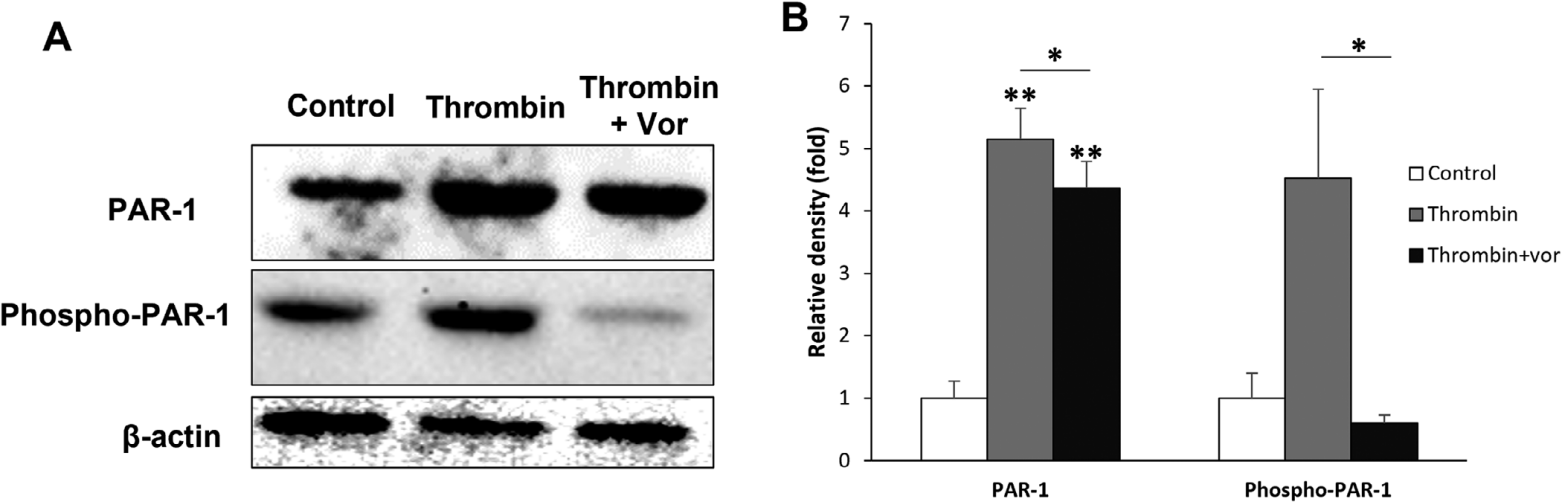
PAR‐1 antagonist suppresses human thrombin‐enhanced phosphorylation of PAR‐1 in HUVECs. HUVECs were treated with 2 U/mL of human thrombin and 100 nM vorapaxar (Vor) for 2 h. Protein was extracted and the expression levels of PAR‐1 and phospho‐PAR1 were determined using western blot analysis. β‐actin was used as an internal control (A). The densitometric analysis of protein bands was obtained by ChemiDoc MP System (B). *p < 0.05, **p < 0.005 versus control group.

PAR‐1 is a high‐affinity receptor for activated thrombin, which stimulates phospholipid hydrolysis by binding to G proteins. The activity of PAR‐1 can be regulated by phosphorylation, intramolecular interactions, and intermolecular interactions [27]. To investigate how thrombin regulates PAR‐1 activation, HUVECs were treated with 2 U/mL of human thrombin and 100 nM of the PAR‐1 inhibitor Vor for 2 h. Proteins were extracted, and the expression levels of PAR‐1 and phosphorylated PAR‐1 were measured by Western blot (Figure 6A). Density analysis of the protein bands indicated that PAR‐1 was upregulated by about 4–5 times by thrombin, regardless of Vor treatment. In contrast, phosphorylated PAR‐1 increased by about 4.5 times only when treated with thrombin, indicating that thrombin promotes PAR‐1 activation through phosphorylation (Figure 6B).

### 3.6 Amino Acid Sequence Incompatibility as a Major Cause of Weak Response of Porcine PAR‐1 to Human Thrombin

**FIGURE 7:**
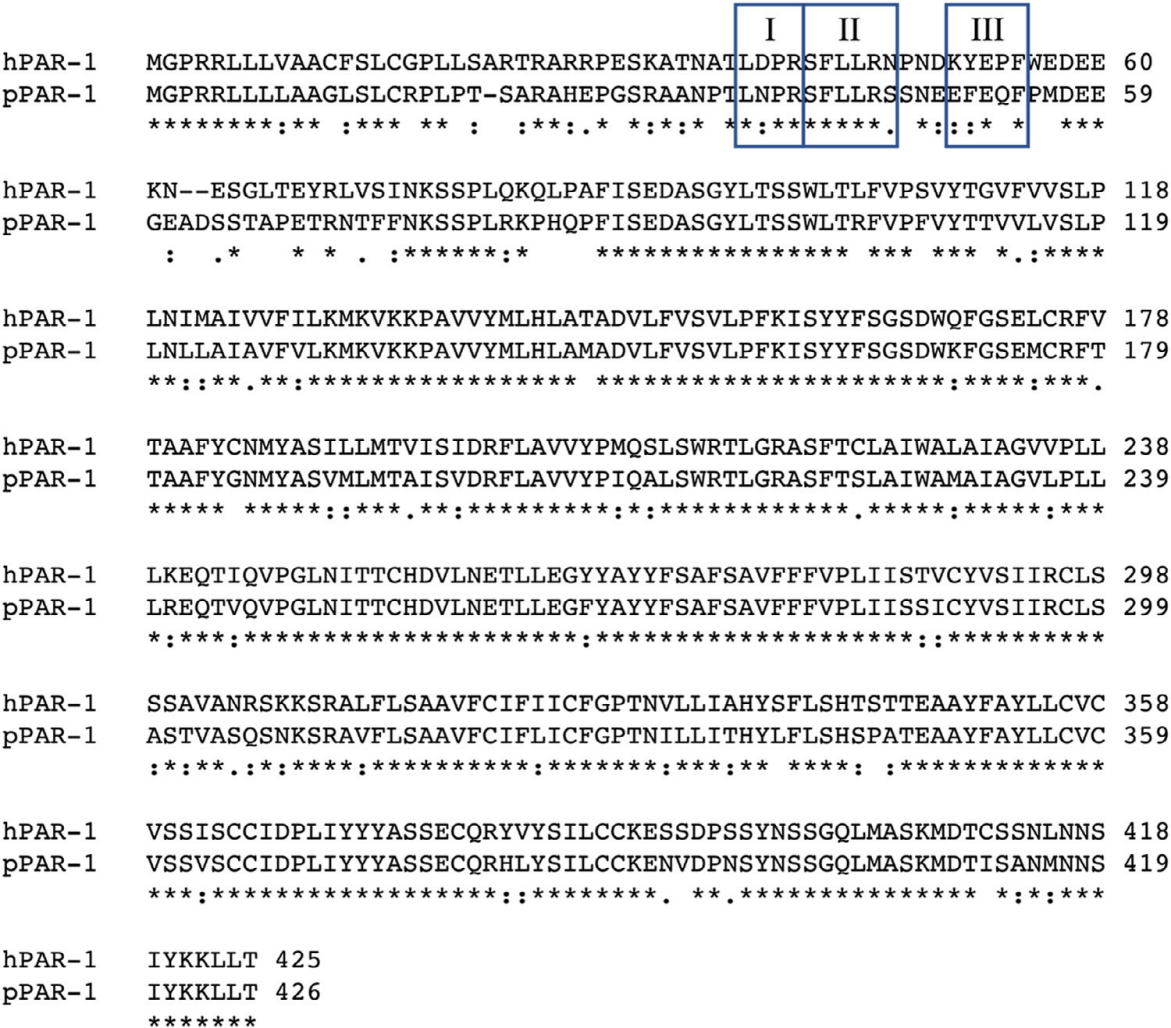
Comparison of PAR‐1 amino acid sequences between human and porcine. The amino acid sequence of human PAR1 (Homo sapiens, Genbank Accession No. NP_001983) is aligned with porcine PAR1 (Sus scrofa, Genbank Accession No. NP_001231301) using Cluster Omega software version 1.2.2. The human PAR1 sequence cleaved by human thrombin (LDPR) and tethered ligand sequence (SFLLRN), hirudin‐like region (KYEPF) are marked in boxes I and II, III, respectively.

Human thrombin activates PAR‐1 by cutting specific amino acids sequence between LDPR^41^ and S^42^FLLRN located N‐terminal exodomain of PAR‐1 [28, 29]. This newly truncated N terminus (S^42^FLLRN) functions as a tethered ligand which activates itself [28]. The N‐terminal exodomain contains a Hir, specifically the sequence K^51^YEPF, which significantly enhances both the specificity and efficiency of thrombin cleavage for PAR‐1 [30]. Steady‐state kinetic measurements with soluble PAR1 N‐terminal exodomains demonstrate the mechanism of thrombin‐PAR‐1 recognition and cleavage which emphasize the essence of the Hir sequence for rapid association with thrombin to form a Hir‐docked complex, subsequently, the L^38^DPR^41^SFL^44^ sequence locks into the active site of thrombin, presenting the highest energy barrier to the overall reaction. Peptide bond cleavage at Arg41‐Ser42 is allosterically enhanced by the docked Hir sequence. Deletion of the K^51^YEPF^55^ Hir sequence or substitution with A^51^AAAK^55^ causes an 8–30‐fold decrease in catalytic efficiency (*k_cat_/K_m_*). Furthermore, the PAR‐1's C‐terminal exodomain product of thrombin cleavage which retains the Hir sequence, corresponding to the activated receptor, binds tightly to thrombin. This would suggest that an additional role of the Hir sequence in the thrombin‐activated receptor is to sequester thrombin to the platelet surface and modulate cleavage of other platelet receptors such as the PAR4 thrombin receptor, which lacks a functional Hir sequence [31]. Too high of an affinity for the PAR1 Hir sequence might be detrimental and could seriously impair the proteolysis of other thrombin substrates and further reduce thrombin turnover [32]. Thus, the macromolecular interactions between thrombin and PAR1 must achieve a balance between specificity and rapid activation. To determine whether the PAR‐1 proteins of humans and pigs are compatible with each other, we analyzed the amino acid sequences using NCBI Protein BLAST (Figure 7). Comparing the full amino acid sequences of the PAR‐1 proteins revealed a 78% match. When considering conservative substitutions of amino acids, which include functionally similar replacements, the match rate increased to 88%. The thrombin cleavage site of human PAR‐1 was LDPR^41^↓S^42^FLLRN (Figure 7 Box I and II) and the thrombin cleavage site of porcine PAR‐1 was VELR^41^↓S^42^FFLRK, showing a 50% concordance rate. Both sequences have the cleavage occurring after an arginine (R) residue, which is consistent with the known specificity of thrombin. The Hir of human PAR‐1 was K^51^YEPF (Figure 7 Box III) and Hir of porcine PAR‐1 was D^51^FEPI, the amino acid sequence are 40% identical.

### 3.7 Differential Binding Stability Between Human and Porcine PAR‐1 With Human Thrombin

**FIGURE 8:**
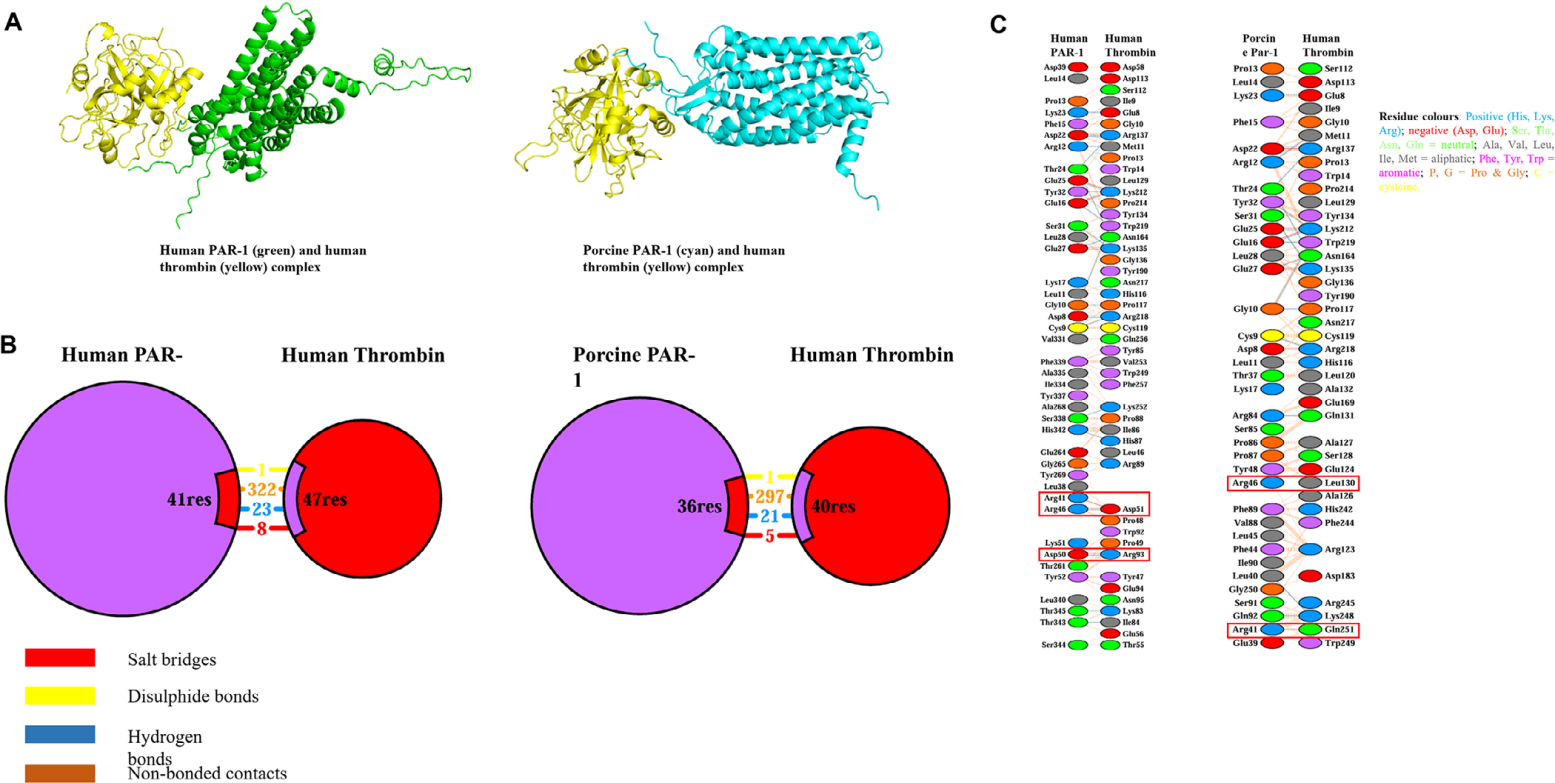
HDOCK‐generated 3D models and PDBsum interaction analysis of human PAR‐1–human thrombin and porcine PAR‐1–human thrombin complexes. (A) Top‐ranked models, showing human PAR‐1 (green) and porcine PAR‐1 (cyan) bound to human thrombin (yellow). (B) PDBsum summary of the types and numbers of interactions within these complexes. (C) Detailed analysis of various interactions, with key interactions highlighted in red.

To better understand how sequence differences affect the binding of human and porcine PAR‐1 to human thrombin, protein‐protein docking and binding models were generated using the HDOCK webserver (Figure 8A,B). Among the 100 models for both complexes, top‐predicted model showed that human PAR‐1 had a sequence coverage and ID of 0.736 and 65.3%, while porcine PAR‐1 had 0.740% and 58.7%; for human thrombin, these were 1.00% and 99.3%. These results, as shown in Table 1, suggest the reliability of the docking analysis, with confidence scores above 0.7 indicating a high likelihood of successful binding. The human PAR‐1 and thrombin complex had a lower docking score than the porcine complex, suggesting greater stability. Interaction analysis using PDBsum revealed that the human complex had 32 interactions, including 8 salt bridges, 1 disulfide bond, and 23 hydrogen bonds, while the porcine complex had 27 interactions (5 salt bridges, 1 disulfide bond, and 21 hydrogen bonds) as detailed in Figure 8B. Key differences include stronger binding at residues R41, R46, and N50 in human PAR‐1, which form additional salt bridges and hydrogen bonds absent in porcine PAR‐1. Human PAR‐1 also formed unique non‐bonded contacts at K51 and Y52, which were absent in porcine PAR‐1. Additionally, porcine PAR‐1 formed hydrogen bonds at A84 and Q92, while human PAR‐1 did so at S338, H342, T343, and T345 (Figure 8C). These differences likely contribute to the distinct binding stability between the two complexes.

**TABLE 1:** Molecular docking and confidence scores for human PAR‐1–human thrombin and porcine PAR‐1–human thrombin complexes generated by HDOCK server.

Protein‐protein complex	Docking score (kcal/mol)	Confidence score
Human PAR‐1 and human thrombin	—272.33	0.9203
Porcine PAR‐1 and human thrombin	—230.76	0.8341

*Note:* Top‐predicted model has the lowest docking score and the highest confidence score. Docking score (kcal/mol): A more negative score indicates a higher possibility of binding. Confidence score: Derived from the docking score, it reflects the likelihood of binding, with values above 0.7 suggesting a high probability of interaction.

## 4 Discussion

Dysregulation of inflammation and coagulation is a major cause of xenotransplantation failure [23], likely due to molecular incompatibilities between species. Porcine vascular endothelial cells do not effectively interact with human coagulation inhibitors, leading to the expression of TF, which activates the coagulation pathway and promotes thrombus formation [3]. Porcine TBM binds to human thrombin but fails to effectively activate protein C, a key anticoagulant, due to structural differences between porcine TBM and human thrombin [3, 33]. These incompatibilities may lead to thrombus formation and subsequent tissue damage in the transplanted organ [3]. Additionally, porcine tissue factor pathway inhibitors (TFPI) are less effective at inhibiting human factor Xa, due to amino acid sequence differences between porcine and human TFPI [34, 35]. Moreover, porcine von Willebrand factor (vWF) spontaneously binds to primate platelet glycoprotein 1b (GP1b) without shear stress, unlike human vWF, leading to platelet aggregation [36]. These findings suggest that molecular incompatibility between porcine and human coagulation‐related molecules may cause thrombosis and tissue damage, ultimately leading to the loss of the transplanted organ. Therefore, overcoming these molecular incompatibilities through genetic modification is essential for successful xenotransplantation. Genetically modified pigs expressing human TBM and other coagulation proteins are already being used in research [37].

Inflammation is another inevitable challenge in solid organ transplantation, often resulting from surgical trauma and ischemia‐reperfusion injury. Damage‐associated molecular patterns released from injured tissues are recognized by innate immune receptors, triggering inflammatory responses that lead to transplant rejection [38, 39]. Suppressing inflammation during the early stages of organ transplantation is crucial for success. However, the inflammatory response in xenotransplantation is much stronger than in allotransplantation [38]. Despite immunosuppressive therapy, systemic inflammation can persist for several months after solid organ xenotransplantation [40, 41]. In addition, the inflammatory environment activates monocytes to express TF, further activating the coagulation response [42, 43, 44]. The reciprocal relationship between inflammation and coagulation amplifies the release of inflammatory mediators and procoagulant factors [45], with PAR‐mediated signaling exacerbating the production of inflammatory cytokines [46]. Therefore, controlling both inflammation and coagulation is critical for successful xenotransplantation.

This study investigated the molecular mechanisms related to immune rejection in xenotransplantation, focusing on the compatibility between porcine PAR‐1 and human thrombin. Our findings suggest that human thrombin does not induce an inflammatory response via porcine vascular endothelial cell PAR‐1, and that the inability of human thrombin to fully activate porcine PAR‐1 is due to amino acid sequence incompatibilities. Human thrombin activates PAR‐1 by cleaving specific N‐terminal sequences, but the sequences upstream of the cleavage site and in the Hir domain of human and porcine PAR‐1 differ significantly. These species‐specific variations likely influence thrombin binding efficiency and kinetics. Post‐cleavage, the tethered ligand sequences also differ slightly (“SFLLRN” in humans vs. “SFFLRK” in pigs), which may affect downstream signaling, leading to differences in receptor activation and cellular responses. Protein‐protein docking analysis revealed that porcine PAR‐1 forms less stable and energetically less favorable interactions with human thrombin compared to human PAR‐1. Protein‐protein interaction analysis further confirmed that the porcine PAR‐1–human thrombin complex exhibits fewer interactions, such as salt bridges and hydrogen bonds, than the human PAR‐1 complex. Specifically, key residues like R41, R46, and N50 in human PAR‐1, which enhance thrombin binding affinity, were found to be crucial for effective cleavage and activation. Additionally, the unique interactions in the Hir domain of human PAR‐1 are absent in porcine PAR‐1, further emphasizing species‐specific differences. These findings suggest reduced thrombin binding and activation efficiency in the porcine system, which could complicate the regulation of blood coagulation and inflammation during porcine‐to‐human organ transplantation.

Experiments using the PAR‐1 inhibitor Vor showed that the expression of inflammatory cytokines and chemokines was suppressed during co‐culture of human macrophages and porcine vascular endothelial cells. This indicates that PAR‐1 plays a critical role in mediating inflammatory responses and that inhibiting PAR‐1 signaling with Vor—a clinically approved PAR‐1 antagonist [47]—could help control inflammation in xenotransplantation. However, Vor is a human‐specific PAR‐1 antagonist designed to target human thrombin–PAR‐1 interactions and is therefore likely to be ineffective in inhibiting porcine thrombin–PAR‐1 signaling [48]. In the present, human thrombin increased Ca^2+^ and NO levels, the expression of inflammatory mediators, and endothelial permeability in HUVECs, but did not induce significant changes in PAOECs. This suggests that human PAR‐1 is activated by human thrombin, whereas porcine PAR‐1 is not, further confirming the molecular incompatibility between these species. Given that thrombin modulates vascular tone by inducing a biphasic response through differential phosphorylation of eNOS via PAR‐1 [15], the molecular incompatibility between human thrombin and porcine PAR‐1, which impairs NO production, signifies the absence of crucial protective factors—such as vasodilation, anti‐inflammatory, and antithrombotic effects—necessary for the survival of the transplanted organ. If vasodilation does not occur properly, the removal of active substances may be delayed, compromising vessel patency, especially in small arterioles, and potentially leading to early failure of the vascularized porcine xenograft. Future studies on xenotransplantation models should include hemodynamic assessments to analyze whether a reduction in NO is associated with vascular complications.

Although modifying porcine PAR‐1 to enhance compatibility with human thrombin could improve endothelial function by increasing thrombin‐induced NO production, thereby enhancing vascular integrity and graft survival, it may also exacerbate immune rejection due to increased inflammatory cytokine production, endothelial activation, and leukocyte infiltration. Thus, fully restoring thrombin–PAR‐1 interactions could be a double‐edged sword. Selective PAR‐1 inhibitors or targeted therapies regulating thrombin–PAR‐1 signaling may provide a more refined approach to improving xenotransplantation outcomes. Additionally, given that Vor has been reported to increase bleeding risk in clinical settings [49] careful regulation of its anti‐inflammatory and anticoagulant effects is necessary when using genetically modified porcine cells expressing human coagulation regulatory factors such as TBM or EPCR.

**FIGURE 9:**
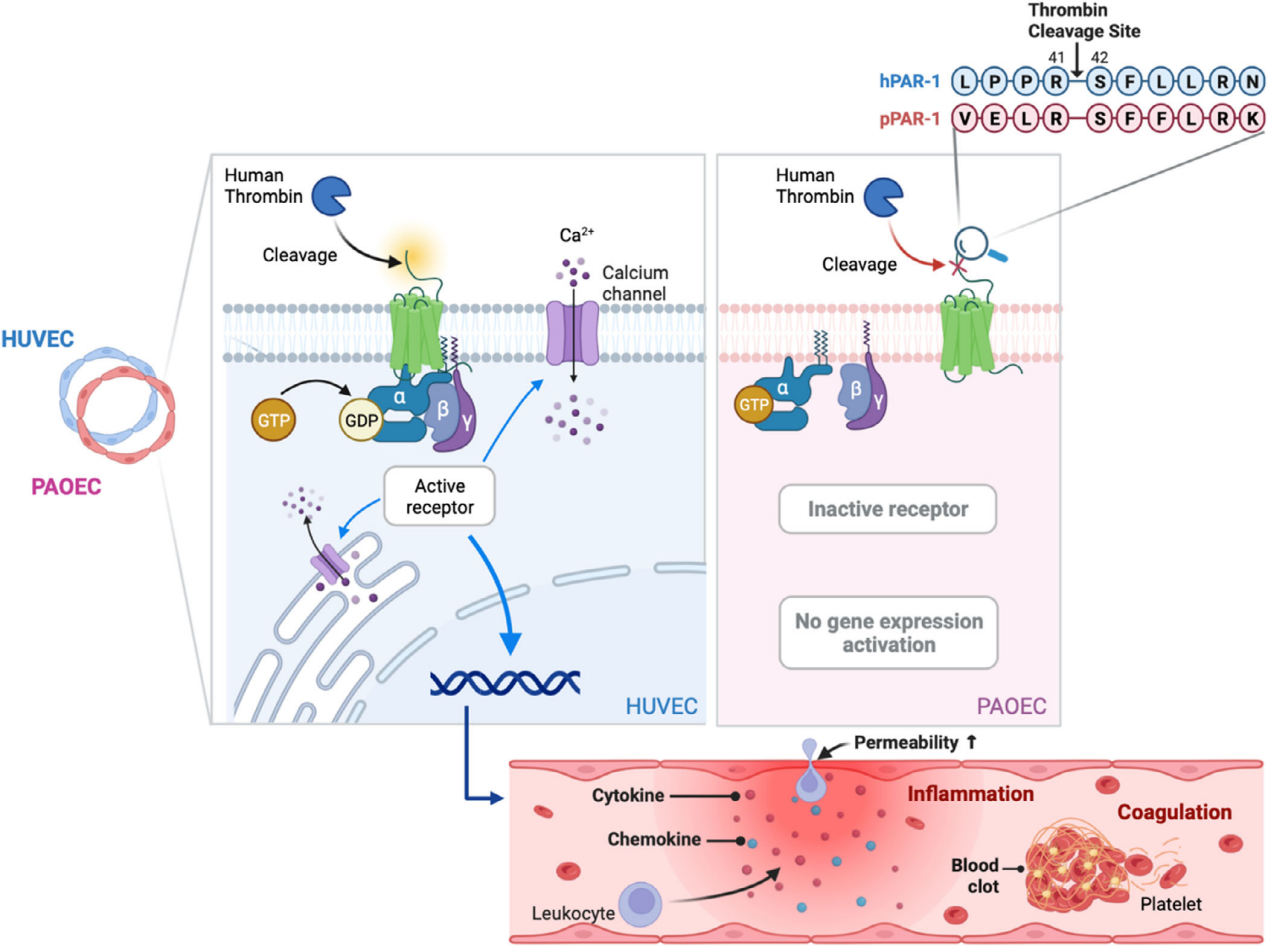
Schematic diagram illustrating differential activation of PAR‐1 by human thrombin in HUVECs and PAOECs. This illustration shows the contrasting effects of human thrombin on HUVECs and PAOECs due to differences in the PAR‐1 thrombin cleavage site. On the left, in HUVECs, human thrombin effectively cleaves and activates PAR‐1 at the cleavage site, leading to receptor activation. This triggers a signaling cascade, including Ca^2+^ influx and gene expression activation, which promotes the production of cytokines and chemokines, increases endothelial permeability, and contributes to inflammation and coagulation. On the right, in PAOECs, due to sequence differences at the thrombin cleavage site, human thrombin does not activate porcine PAR‐1. As a result, the receptor remains inactive, preventing gene expression activation and subsequent cellular responses. This species‐specific interaction underlines the molecular incompatibility between porcine PAR‐1 and human thrombin, which could impact xenotransplantation outcomes.

In conclusion, this study provides critical insights into the molecular mechanisms of immune rejection during xenotransplantation between humans and pigs, confirming for the first time that structural differences in PAR‐1 significantly influence its binding to human thrombin. The study identified unique structural features—resulting from differences in amino acid sequences and interaction types—that are crucial for this binding and the subsequent receptor activation, which triggers a signaling cascade, including Ca^2+^ influx, gene expression, and increased endothelial permeability (Figure 9). As PAR‐1 regulates both coagulation and inflammatory responses, modifying porcine PAR‐1 to enhance compatibility with human thrombin or using PAR‐1 inhibitors to address inflammatory responses resulting from interactions between human macrophages and porcine endothelial cells may improve xenotransplantation outcomes. A limitation of this study is that the experiments were conducted in vitro, which may not fully reflect the complex in vivo physiological and immunological environment. Future research should verify these predicted binding models through mutagenesis and other experimental techniques, and clarify the functional consequences of these differences, ultimately contributing to the development of more effective therapeutics targeting thrombin‐PAR‐1 interactions. These studies could significantly improve the success rates of xenotransplantation.

## Ethics Statement

No ethics approval was required for this work as it was performed in vitro using a cell line and no primary human samples were used.

## Consent

All authors consent to the publication of this study.

## Conflicts of Interest

The authors declare no conflicts of interest.
